# The near-infrared bacteriophytochrome-derived fluorescent protein PENELOPE enables RESOLFT superresolution microscopy

**DOI:** 10.1073/pnas.2504748122

**Published:** 2025-11-24

**Authors:** Daniel Stumpf, Nickels Jensen, Cédric Mittelheisser, Jan Keller-Findeisen, Alexey I. Chizhik, Maria Kamper, Timo Diekmann, Florian Habenstein, Isabelle Jansen, Jörg Enderlein, Michel Sliwa, Kaushik Inamdar, Stefan W. Hell, Stefan Jakobs

**Affiliations:** ^a^Department of NanoBiophotonics, Max Planck Institute for Multidisciplinary Sciences, Göttingen 37077, Germany; ^b^Laboratoire de Spectroscopie pour les Interactions, la Réactivité et l’Environnement, UMR 8516, Université de Lille CNRS, Lille 59000, France; ^c^Fraunhofer Institute for Translational Medicine and Pharmacology, Translational Neuroinflammation and Automated Microscopy, Göttingen 37075, Germany; ^d^Institute of Physics, Georg August University, Göttingen 37077, Germany; ^e^Cluster of Excellence “Multiscale Bioimaging: from Molecular Machines to Networks of Excitable Cells”, University of Göttingen, Göttingen 37099, Germany; ^f^Laboratoire d’Optique et Biosciences, CNRS, INSERM, École Polytechnique, Institut Polytechnique de Paris, Palaiseau 91120, France; ^g^Clinic of Neurology, University of Göttingen, Göttingen 37075, Germany

**Keywords:** superresolution microscopy, RESOLFT, fluorescent proteins, live-cell imaging

## Abstract

REversible Saturable Optical Linear Fluorescence Transitions (RESOLFT) superresolution microscopy fundamentally overcomes the diffraction barrier in far-field fluorescence microscopy. It relies on reversibly switchable fluorescent proteins (RSFPs) that allow repeated light-induced transitions between fluorescent on- and nonfluorescent off-states. Because these transitions are induced by low-light intensities, RESOLFT superresolution microscopy is particularly suitable for live-cell imaging. So far, RESOLFT imaging has only been performed in the visible range of the electromagnetic spectrum. To expand the RESOLFT concept into the near-infrared (NIR) region, which is characterized by reduced autofluorescence, lower scattering and decreased phototoxicity, we developed the **p**hotostabl**e N**IR r**e**versibly switchab**l**e flu**o**rescent **p**rot**e**in (PENELOPE), which is the first RSFP applicable in the NIR window. PENELOPE was generated by mutagenesis of the chromophore-binding domain of the *Deinococcus radiodurans* bacteriophytochrome. This NIR-RSFP exhibits high photostability and high ensemble switching contrast at low-light intensities. It also undergoes an unusually fast thermal fluorescence recovery from the dark state into an on-state. This was exploited for low-light intensity RESOLFT imaging with only a single wavelength, as the same light wavelength (660 nm) is used for off-switching and fluorescence readout, while the on-switching occurs in the absence of illumination. We demonstrate RESOLFT recordings both in chemically fixed and in living human cells using PENELOPE as a fusion protein.

## Significance

Low-light REversible Saturable Optical Linear Fluorescence Transitions (RESOLFT) nanoscopy is particularly suitable for live-cell imaging. However, currently available reversibly switchable fluorescent proteins (RSFPs) emitting in the visible spectrum require potentially phototoxic short-wavelength switching, restricting their use in living cells. To address this, we developed the bacteriophytochrome-derived near-infrared (NIR)-RSFP photostable NIR reversibly switchable fluorescent protein (PENELOPE). This protein thermally relaxes to a fluorescent on-state in milliseconds, allowing the implementation of single-wavelength NIR-RESOLFT imaging. We show the compatibility of PENELOPE with mammalian cell expression and demonstrate subdiffraction RESOLFT imaging in living and fixed human cells. Taken together, we present a new RSFP, PENELOPE, which integrates the flexibility of a protein tag having the advantages of NIR imaging with live-cell RESOLFT nanoscopy.

Subdiffraction superresolution microscopy, or nanoscopy, fundamentally overcomes the diffraction limit in fluorescence microscopy. Conceptually, most nanoscopy variants switch off the fluorescence ability of the majority of the fluorophores within a diffraction region and record few or even single on-state molecules at a time (1). All these methods for cellular imaging, thus, rely on molecular transitions (switching) of molecules between two states, typically a fluorescent on-state and a nonfluorescent off-state (2, 3).

In coordinate-targeted nanoscopy methods such as STimulated Emission Depletion (STED) (4, 5) or REversible Saturable Optical Linear Fluorescence Transitions (RESOLFT) (6–8), light is used to induce transitions between a fluorescent on-state and a nonfluorescent off-state at defined coordinates (1). Most coordinate-targeted nanoscopy implementations rely on point-scanning; i.e., a doughnut-shaped light focus featuring a minimum (zero) at its center is scanned over the sample. At the light intensity zero, no off-switching occurs, and the fluorophores in the on-state fluoresce upon irradiation with light of the respective readout wavelength.

RESOLFT nanoscopy stands out from all other nanoscopy methods, as it requires very low-light intensities to overcome the diffraction barrier (9–12), rendering it particularly attractive for live-cell recordings. In general, RESOLFT imaging requires reversibly switchable fluorescent proteins (RSFPs) that exhibit metastable on- and off-states. Recently, RESOLFT imaging has also been reported in conjunction with switchable organic fluorophores (13, 14).

So far, all reported RSPSs emit in the visible part of the spectrum and have been derived from fluorescent proteins isolated from hydrozoa, sea anemones, or stony corals (15, 16). Importantly, all of these RSFPs need to be switched in one direction with UV-light or violet light. As short light wavelengths are prone to induce phototoxic effects (17), this property partly counteracts the advantage of RESOLFT imaging being particularly benign for live-cell imaging.

The near-infrared (NIR) spectral illumination range of 650 nm to 900 nm is ideal for live-cell imaging (18). It offers beneficial properties in terms of reduced phototoxicity (19), decreased autofluorescence, increased penetration depth in vivo (20), as well as additional possibilities for spectral multiplexing (21).

In this study, we set out to establish RESOLFT imaging of living cells using wavelengths entirely in the NIR spectral regime. As no GFP-like fluorescent proteins with excitation maxima above 650 nm exist (22), we decided to rely on bacteriophytochromes as a template to develop a NIR switchable fluorophore (23). In contrast to GFP-like fluorescent proteins, bacteriophytochromes do not autocatalytically form a chromophore, but rely on the linear tetrapyrrole biliverdin (BV) as an external chromophore. In most eukaryotic cells, BV is part of the heme-degradation cycle (24) and is therefore ubiquitously present in mammalian cells.

Naturally occurring bacteriophytochrome holoproteins function as light sensors and can be repeatedly converted between a red light-absorbing form (Pr-form) and a far-red light-absorbing form (Pfr-form), acting as photo sensors for signal transduction. The Pr-Pfr transition involves several micro- and milliseconds intermediate species (25). Interconversion between these two forms can be achieved by irradiation with red/far-red light or by thermal relaxation in the dark to the Pr ground state or to the Pfr ground state in the case of bathy phytochromes (26). This interconversion can be exploited to generate reversible switchable fluorescent proteins. The Pr-form of bacteriophytochrome-derived fluorescent proteins emits upon absorption of red light, thus functioning as the fluorescent on-state in the context of red-light illumination, while their Pfr-form functions as a dark off-state with a low fluorescence quantum yield (QY). Several approaches to engineer bacteriophytochrome-derived fluorescent proteins aimed to suppress the inherent Pr-Pfr photoconversion of bacteriophytochromes to generate bacteriophytochromes trapped in a fluorescent Pr-state (27, 28). These strategies yielded constitutively active fluorescent proteins such as mIFP or mIRFP670, which can serve as genetically encoded markers for imaging cells and organisms in the NIR range (27, 29) and have found applications in STED nanoscopy (30, 31). In addition, photoconverting bacteriophytochrome-derived proteins (miRFP mutants from *Rhodopseudomonas palustris*) have also been demonstrated as being compatible with in vivo STED imaging (32).

However, there are currently no reversibly switchable bacteriophytochrome-derived fluorescent proteins for RESOLFT-type superresolution microscopy applications. The only reported photomodulatable bacteriophytochrome-derived fluorescent proteins compatible with cellular imaging are the photoactivatable PAiRFP1 and PAiRFP2 (25, 33). But these proteins have low effective brightness, making them unsuitable for use in superresolution microscopy.

In this study, we decided to take a different approach in designing a bacteriophytochrome derivative for the application in RESOLFT superresolution microscopy: Instead of combating the switching and stabilizing the fluorescent Pr-state, we aimed to maintain the natural switching tendency from the fluorescent Pr (on) state to the nonfluorescent Pfr (off) state, while increasing the fluorescence brightness of the on-state in order to generate a switchable bacteriophytochrome for the application in RESOLFT imaging. To this end, the bacteriophytochrome-derived NIR-RSFP PENELOPE was engineered. The PENELOPE on-state is excited by light of 660 nm, the emission occurs at 720 nm, and the protein is switched to a nonfluorescent off-state. Additionally, while PENELOPE can be transferred back to the fluorescent on-state by applying 785 nm laser light, it also exhibits fluorescence recovery by thermal relaxation in the order of milliseconds from the off- to the on-state in living cells. This feature allowed us to develop a single wavelength imaging regime without the need for an on-switching laser enabling low-light level RESOLFT imaging in the NIR spectrum in living cells.

## Results

### Development of a Bacteriophytochrome-Derived NIR-RSFP.

For the purpose of generating a NIR-RSFP for RESOLFT imaging, we turned to the monomerized chromophore-binding domain (CBD) consisting of the PAS and GAF domains of the *Deinococcus radiodurans* bacteriophytochrome (*SI Appendix*, Fig. S1). Several constitutively fluorescent NIR-FPs, including WiPhy (34), IFP2.0 (35), and SNIFP (30), have been successfully generated based on this template. In addition, high-resolution crystal structures of WiPhy and IFP2.0 were available (PDB 3S7Q, 4O8G) (34, 36). In a previous study, during the engineering of the constitutively fluorescent SNIFP (30), we had identified a CBD variant comprising 331 amino acids (Dr-CBD_mono_, denoted as W3 in that study). This W3 variant contained an extra 10 amino acids at its C-terminus relative to the SNIFP construct, which preserved rudimentary switching capabilities, while exhibiting high molecular brightness. We decided to use this variant as a starting template in the search for improved reversibly switchable variants.

**Fig. 1.**
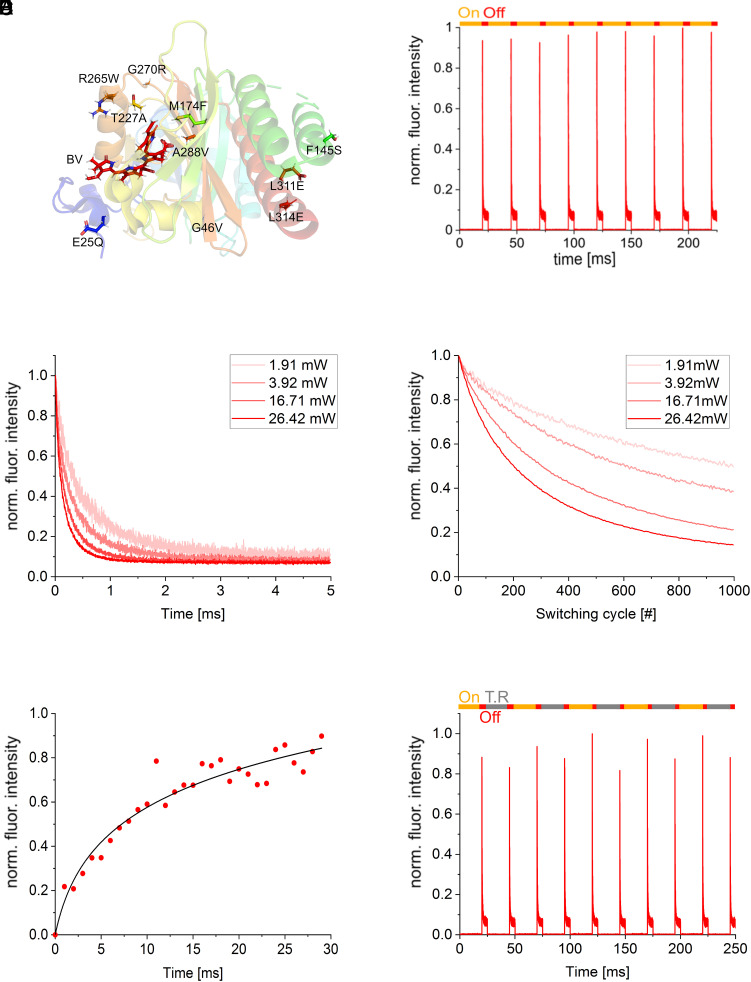
Switching characteristics of PENELOPE in living bacteria. (*A*) Structure of the monomeric infrared fluorescent *D. radiodurans* bacteriophytochrome CBD [PDB ID: 3S7Q; (34)]. Labels indicate mutations introduced to generate PENELOPE. (*B*) Normalized multicycle fluorescence traces of PENELOPE. Switching steps are indicated with a color code above the fluorescence traces. On-switching steps with light of 785 nm are indicated in orange, and off-switching and fluorescence excitation with light of 660 nm are indicated in red. On-switching was performed for 20 ms with a power of 14.32 mW, and off-switching was performed for 5 ms with a power of 10.67 mW (all powers are measured behind the objective). (*C*) Single fluorescence traces of the off-switching using different laser powers. (*D*) Switching fatigue as number of switching cycles in bacterial colonies. Line graphs represent maximum fluorescence intensities of the off-switching curves of every cycle. (*E*) Normalized thermal relaxation of PENELOPE. Data points were fitted with a logarithmic function. PENELOPE had been switched to 10% residual fluorescence intensity in the ensemble off-state before relaxation. (*F*) Normalized multicycle fluorescence traces of PENELOPE. Every other light-driven on-switching step is replaced for an illumination-free thermal relaxation step. Switching steps are indicated with a color code above the fluorescence traces. On-switching steps with light of 785 nm are indicated in orange, off-switching and fluorescence excitation with light of 660 nm is indicated in red, and the nonilluminated thermal relaxation step is indicated in gray. On-switching, with a power of 14.32 mW was performed for 20 ms. Allowed thermal relaxation interval was also 20 ms. Off-switching was performed for 5 ms with a power of 10.67 mW.

This W3 variant had been optimized for mammalian codon usage and already incorporated three previously reported mutations ensuring monomerization (F145S, L311E, and L314E) (30, 34) (Fig. 1*A* and *SI Appendix*, Fig. S2). This monomerized and codon-optimized *D. radiodurans* CBD-F145S, L311E, L314E variant exhibited switching upon irradiation with light of 660 nm and 785 nm for off- and on-switching, respectively (Table 1), but had a poor switching contrast.

**Table 1.** Photophysical properties of PENELOPE in comparison to constitutively fluorescent NIR-FPs and the precursor Dr-bacteriophytochrome

Photophysical characteristics		mIRFP670^*^	Dr-CBD_mono_ (W3)	SNIFP^†^	PENELOPE
Absorption max [nm]	on-state, pure Pr (Q-band)^‡^	644	696	690	689
	off-state (Q-band)	na	742	na	691
Excitation max [nm]	eq, pH 7.5 (Q-band)	642	nd	697	690
Emission max [nm]	eq, pH 7.5 (Q-band)	670	720	720	720
EC at abs. max [M^–1^ cm^–1^] of holo-protein	eq-state, pH 7.5	87,400	67,500	149,200	86,100
Fluorescence QY (eq-state)	eq-state, pH 7.5	0.14^§^	na	0.022^§^	0.09^**^
Molecular brightness^††^	eq-state, pH 7.5	12.2	na	3.28	7.75
Off-Switching QY (Off-QY)	on-state, pH 7.5	na	0.05	na	0.03
On-Switching QY (On-QY)	off-state, pH 7.5	na	0.03	na	0.01
Fluorescence lifetime [ns]	in solution, pH 7.5	nd	na	0.63	0.69
Residual fluorescence intensity in the ensemble off-state [% of on-state]	bacterial colonies	na	na	na	6.08
Off-Switching half-time [µs]	bacterial colonies	na	na	na	86
No. of switching cycles to reach 50% maximal intensity	bacterial colonies	na	na	na	972
Thermal recovery [s]	In solution pH 7.5, 20 °C	na	6.5 × 10^2^ (37%) 4.0 × 10^3^ (45%) 1.3 × 10^4^ (18%)	na	5.8 × 10^2^ (22%) 1.6 × 10^4^ (78%)
	In *E. coli* PBS, 20 °C	na	nd	na	6.8 × 10^1^ (3%) 7.7 × 10^2^ (97%)
	In *E. coli* PBS, 37 °C	na	nd	na	1.2 × 10^1^ (1%) 3.6 × 10^2^ (99%)

^*^From ref. 27.

^†^From ref. 30.

^‡^Switched on-state.

^§^Relative QY determination via fluorescence spectra.

^**^Absolute QY determination using a plasmonic nanocavity.

^††^Product of EC and QY divided by 1,000.

To improve the switching characteristics further, a semirational mutagenesis approach was selected, and mutant libraries were screened for minimal residual fluorescence in the nonfluorescent off-state, cellular brightness, switching speed as well as resistance to switching fatigue. For the screening of these parameters, we utilized an automated custom-built fluorescence screening microscope. The screening microscope allowed to illuminate bacterial colonies expressing fluorescent proteins in a preprogrammed manner. Initially, we screened for variants for which fluorescence could be excited with light of 660 nm and simultaneously allowed switching from a fluorescent on-state to a nonfluorescent off-state. Switching back from the nonfluorescent off-state to the fluorescent on-state was expected to be achieved by illumination with light of 785 nm. We chose these wavelengths as they lie within the NIR window at the expected absorbance peaks of the respective Pr- and Pfr-states. Also, suitable CW-laser sources are readily available, and respective laser powers were chosen to enable switching in *Escherichia coli* colonies on a microsecond to millisecond time range.

In order to generate plasmid libraries of suitable variants, we performed extensive random mutagenesis as well as directed mutagenesis on a plasmid encoding CBD-F145S, L311E, L314E. First, several rounds of site-directed mutagenesis were employed with the aim to change amino acids in close proximity to the D-ring of the BV chromophore. The plasmid libraries were transformed in *E. coli* cells that were cotransformed with a plasmid expressing a *Bradyrhizobium* heme-oxygenase to ensure sufficiently high levels of BV. We prioritized variants with a low residual fluorescence, as this parameter is fundamental for the generation of RESOLFT superresolution images. Using site-directed mutagenesis, we identified the mutations E25Q, M174F, and A288V, which increased the brightness of the fluorescent on-state and decreased the residual fluorescence in the off-state.

Subsequently, 10 rounds of random mutagenesis were performed. Random mutagenesis aimed at the improvement of switching speed, resistance to switching fatigue, and photostability. Thereby, we identified the additional mutations G46V, R265W, T227A, and G270R, all of which were located outside the chromophore-binding pocket. In these consecutive screening rounds, altogether ~10,000 individual clones were analyzed. The resulting final variant was CBD E25Q, G46V, F145S, M174F, T227A, R265W, G270R, A288V, L311E, L314E (Fig. 1*A* and *SI Appendix*, Fig. S2). This new bacteriophytochrome-derived NIR-RSFP was named PENELOPE for **p**hotostabl**e N**IR r**e**versibly switchab**l**e flu**o**rescent **p**rot**e**in.

Together, we generated a new bacteriophytochrome variant, that differs from the original bacteriophytochrome CBD sequence (W3) by ten mutations. It exhibits increased molecular brightness in the on-state while maintaining its rapid switchability and low residual fluorescence in the off-state. PENELOPE can be excited by light of 660 nm and simultaneously switched from a fluorescent on-state to a nonfluorescent off-state by the same wavelength.

### Photophysical and Biochemical Properties of PENELOPE.

To characterize the switching capabilities of PENELOPE in more detail, we first expressed the protein in *E. coli* and characterized its properties by using different irradiation schemes on the living bacteria using laser light of 660 nm and 785 nm for switching.

PENELOPE is a negative reversibly switching NIR-FP that can be switched repeatedly between a fluorescent on-state and a nonfluorescent off-state (Fig. 1*B*). The off-switching kinetics were power dependent (Fig. 1*C*). At 26.42 mW of 660 nm light measured in the sample plane in front of the objective, a switching half-time of 86 ± 4 µs and a minimum residual fluorescence of 6.08 ± 0.29% were observed (Table 1, Fig. 1*C*, and *SI Appendix*, Fig. S3*A*). In *E. coli*, PENELOPE exhibited pronounced resistance to switching fatigue, facilitating about 1,000 cycles between the fluorescent on-state and the nonfluorescent off-state at 1.91 mW of 660 nm, before its brightness diminished to half of its initial intensity (Fig. 1*D* and *SI Appendix*, Fig. S3*B*).

Next, we aimed at characterizing the thermal relaxation kinetics of PENELOPE. This was first performed by measuring the recovery in bulk absorption rates with purified protein and living bacterial cells. Here, we found that purified PENELOPE in protein buffer at pH 7.5 exhibited absorption recovery without illumination (thus attributable to thermal relaxation) in the range of hours (~4.4 h for 78% recovery), in contrast to the faster recovery observed in living bacteria in suspension (6 to 12 min for 98% recovery) (Table 1 and *SI Appendix*, Fig. S3*H*). We also observed a temperature-dependent acceleration of absorption recovery in these living cells (*E. coli*, at 20 °C vs. 37 °C, Table 1 and *SI Appendix*, Fig. S3*H*). The steps of interconversion through several intermediate species have been reported to be strongly sensitive to changes in local environment (37) and could explain the faster recovery of PENELOPE in the intracellular environments of living cells vs. the slower recovery in vitro.

Following this, we focused on measuring thermal-relaxation-induced fluorescence recovery of PENELOPE. For this purpose, we implemented a switching scheme on living bacteria that comprised the same off-switching and fluorescence readout settings induced by light of 660 nm as used for determining switching, but alternated between 785 nm light-driven on-switching, and nonilluminated thermal relaxation-driven recovery from the off-state to an on-state for the same time period (Fig. 1*F*). Interestingly, this thermal relaxation under dark conditions appeared to induce a much faster measured fluorescence recovery than the observed absorption recovery. We found that PENELOPE exhibited recovery from a nonfluorescent off-state to a fluorescent on-state in living bacteria within milliseconds without 785 nm illumination (Fig. 1*E*). Kinetics of fluorescence intensities with light-independent thermal relaxation show that this recovery had a logarithmic growth curve, reaching 50% of its fluorescence brightness at thermal equilibrium after 6.8 ms, with recovery slowing down at longer time scales (Fig. 1*E*). Yet, the fluorescence recovery profiles of light-driven on-switching and thermal relaxation-based on-switching were different. The on-state recovery without illumination (thermal relaxation) consistently resulted in 80 to 85% of the equilibrium fluorescence, at the same time scales as light-driven recovery (90 to 95%) (Fig. 1 *E* and *F*). Based on molecular dynamics simulations, the Pfr state of the *D. radiodurans* has been proposed to have a higher degree of thermodynamic freedom with a microsecond interplay between two possible metastable states (38). In addition, a Pfr-specific intermediate state is seen during thermal relaxation, indicating that this phytochrome thermally relaxes via a different pathway as compared to the light-driven reversion (38), possibly leading to subtle distinctions in the final Pr state. This could explain the observed differences in the two modes of on-switching. However, the differences in final fluorescence intensities of the two modes, though consistent, were only ~10 to 15%. Hence, it can be concluded that the fluorescence recovery rates for light-driven switching and dark recovery are similar over this time interval. In both cases (i.e., with and without illumination for the off- to on-state transition), the recovered fluorescence intensities were sufficiently high to provide a switching contrast potentially suitable for imaging.

This high switching contrast upon recovery taken together with the rapid off-switching kinetics, the minimal residual fluorescence, and the strong resistance to switching fatigue, rendered PENELOPE a promising candidate for RESOLFT imaging. In addition, the fast fluorescence recovery without requiring an on-switching illumination step created the possibility of a single-wavelength imaging regime.

We next aimed at characterizing the spectral properties of PENELOPE in depth, starting with the purified protein in buffer (100 mM Tris-HCl, 150 mM NaCl, pH 7.5). Purified PENELOPE in the light-induced on-state as well as in thermal equilibrium showed a fluorescence excitation maximum at 690 nm and an emission maximum at 720 nm. Samples in thermal equilibrium were completely shielded from light until measurement. Purified PENELOPE exhibited three absorption maxima at 278 nm, 391 nm, and 689 nm, representing the absorption maxima of aromatic amino acids, the Soret band the Q-band of the protein-bound biliverdin (BV), respectively (Table 1 and *SI Appendix*, Fig. S3 *C* and *D*). Since expression and purification may result in a mixture of BV-bound and unbound protein, we quantified the proportions of BV-bound holo-protein and the unbound apo-protein; This ratio of holo- to apo-protein was 1:2.2, indicating that ~31% of PENELOPE was holo-protein i.e., BV-bound (*SI Appendix*, Table S2). As the entire ε(λ) spectrum is anchored at the BV Soret maximum by defining ε391 nm = 39,900 M^−1^cm^−1^per holo-protein (i.e., per BV chromophore), the extinction coefficient (EC) values would be valid for the holo-protein i.e., the BV-bound PENELOPE chromophore. The measured EC at the Q-band absorption maximum (689 nm) for the BV-bound holoprotein was thus 86,100 M^–1^cm^–1^ (Table 1 and *SI Appendix*, Table S1). When exposed to 660 nm light (inducing the transfer to the off-state), the absorption band at 689 nm diminished, while a shifted absorption band emerged at 720 nm (*SI Appendix*, Fig. S3 *C* and *D*). In addition, the Soret band absorption increased. We conclude that the off-state is a mixture of Pr and Pfr forms. Their respective concentrations were determined using fluorescence switching contrast allowing to determine a pure Pfr absorption spectrum (*SI Appendix*, Fig. S3*C*).

The p*K*_a_ of PENELOPE at thermal equilibrium was found to be 4.5 (*SI Appendix*, Fig. S3*G*). With high Q-band absorptions at pH 5.5, pH 7, and pH 8, PENELOPE exhibits favorable resistance to pH values found within organelles and compartments in mammalian cells.

The fluorescence quantum yield of 0.09 of PENELOPE at thermal equilibrium was determined using a plasmonic nanocavity, a method that provides an absolute value of the fluorescence quantum yield (39). Accordingly, the molecular brightness of PENELOPE was 7.75 (being the product of the EC and the fluorescence quantum yield, divided by 1,000) (Table 1). We, thus, engineered PENELOPE aiming for a higher fluorescence quantum yield and a more efficient fluorescent pathway. However, this may have led to a more constrained chromophore potentially increasing the energy barrier for the isomerization-based switching, thus reducing the switching yield. Fully in line with this assumption, we found that the switching quantum yields for off- and on-switching of PENELOPE were lower (Off-QY 0.03, On-QY 0.01) than those of its precursor Dr-CBD_mono_ (Off-QY 0.05, On-QY 0.03) (Table 1).

### PENELOPE as a Tag for Live-Cell Imaging.

**Fig. 2.**
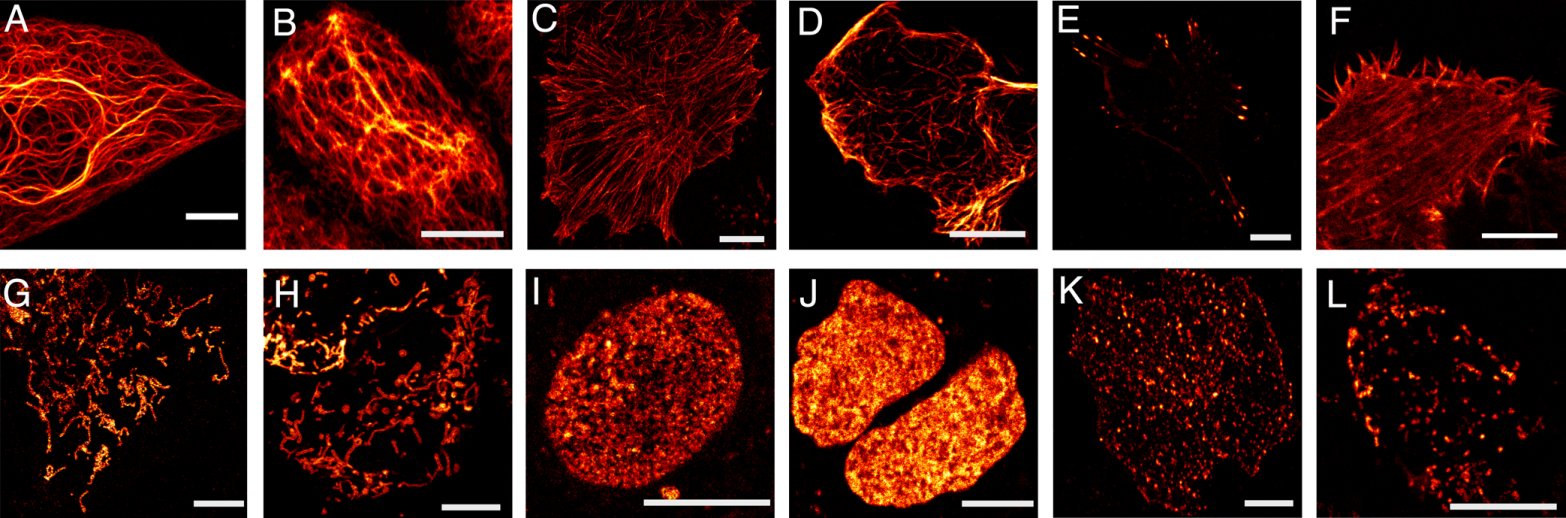
Confocal images of living HeLa cells expressing PENELOPE fusion proteins. Cells were incubated with 25 µM BV for 2 h prior to imaging (*A*) Cytokeratin-18, (*B*) Vimentin, (*C*) Microtubules (α-Tubulin), (*D*) Microtubule ends (EB3), (*E*) Zyxin, (*F*) Actin (LifeAct), (*G*) Mitochondria (Omp25), (*H*) Mitochondria (Cox8a), (*I*) Nuclear pores (Nup50) (*J*) Histones (Histone H2B type 1-N), (*K*) Lysosomes (Lamp1) and (*L*) Peroxisomes. Background subtraction was performed on (*D*, *E*, *I*, *K*, and *L*). (Scale bar: 10 μm.)

Upon seminative SDS PAGE as well as in size exclusion chromatography, PENELOPE behaved as a monomer (*SI Appendix*, Fig. S4). In order to test PENELOPE’s usefulness as a translational tag in NIR live-cell microscopy, it was fused to several host proteins targeted to various cellular structures including cytoskeletal elements (keratin, vimentin, α-tubulin, microtubule ends, zyxin and F-actin), mitochondria (outer and inner membrane), nuclear pores, histones, lysosomes, and peroxisomes in human HeLa cells (Fig. 2). All expressed fusion proteins labeled the expected cellular structures, demonstrating that PENELOPE was suitable as a fluorescent protein tag for expression and imaging in mammalian cells.

**Fig. 3.**
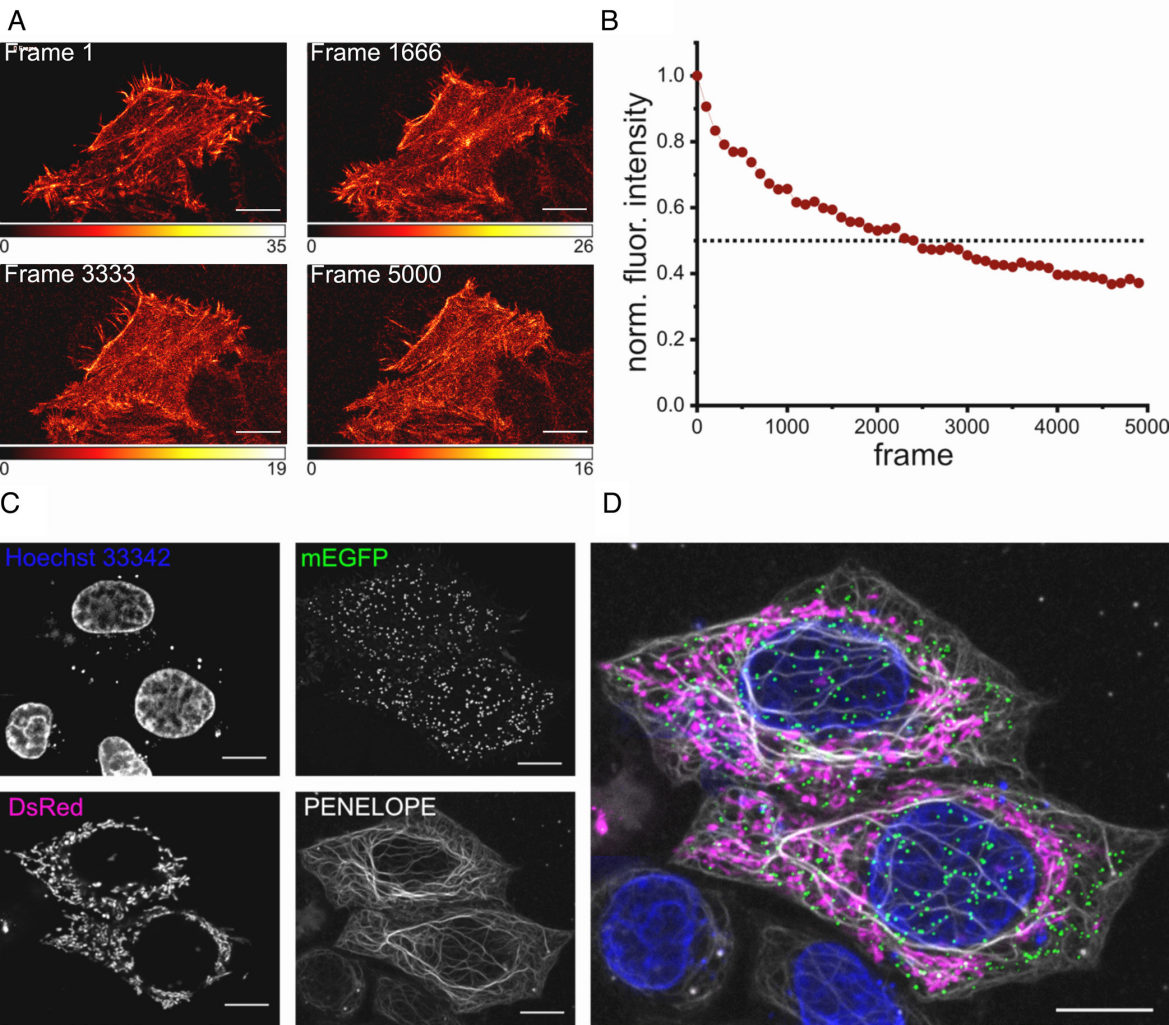
Applications of PENELOPE in confocal imaging. (*A*) Photobleaching of PENELOPE under confocal conditions. 5,000 consecutive images of transiently transfected live HeLa cells expressing LifeAct-PENELOPE were recorded. The presented images display selected frames of the recorded time-lapse measurement. For better visualization, a bleaching correction based on a histogram matching algorithm (40) was applied. (*B*) Normalized fluorescence intensity of the raw images plotted against the frame number. The fluorescence intensity was calculated as an average of the selected ROI, and every 100th value was plotted. (*C*) Single color maximum intensity projections of confocal optical sections of HeLa cells stably expressing cytokeratin-18-PENELOPE. Nuclear DNA was stained with Hoechst 33342. Cells were transfected with lysosome-mEGFP to visualize lysosomes and with pMito-DsRed to visualize mitochondria. (*D*) Merged multicolor image showing nuclear DNA (blue), lysosomes (green), mitochondria (magenta), and cytokeratin-18 (gray). (Scale bar: 10 µm.)

To investigate the photostability of PENELOPE in living cells, we transiently expressed PENELOPE fused to LifeAct targeting the actin cytoskeleton. 24 h after transfection, a single cell was imaged continually with a confocal microscope for ~15 h, resulting in 5,000 image frames (Fig. 3*A*). To better assess the influence of the prolonged imaging on image quality and cellular integrity, we corrected the recorded images for bleaching using a histogram matching algorithm (40). Even after 5,000 image frames, the actin cytoskeleton was clearly delineated, and no signs of cellular stress were observed. After ~2,300 frames, the average image brightness was still 50% of the initial brightness, demonstrating the pronounced bleaching resistance of PENELOPE (Fig. 3*B*).

An additional benefit of using fluorescent probes emitting in the NIR window is the possibility of multiplexing with fluorophores emitting in the visible spectrum. To demonstrate this, we developed a cell line stably expressing PENELOPE-tagged cytokeratin-18, and imaged it in living cells together with Hoechst 33342 and transiently expressed mEGFP and DsRed, targeted to the lysosome and the mitochondria, respectively (Fig. 3*C*). Thereby, these living cells were labeled by four different fluorophores emitting in the blue, green, red, and NIR; multicolor imaging was performed without any postprocessing to separate the four channels (Fig. 3*D*), while proving the utility of PENELOPE as a stable tag for mammalian cells.

### Switching Characteristics of PENELOPE in Mammalian Cells.

Next, we characterized the switching kinetics of PENELOPE in living as well as fixed mammalian cells in a confocal microscope to establish a RESOLFT protocol for nanoscopy in the NIR window. To this end, confocal images of live mammalian cells expressing cytokeratin-18-PENELOPE were taken using different illumination intensities, and at every pixel, the time resolved fluorescence intensity was measured for 3 ms. For the analysis of the kinetic switching curves, intensity-threshold-dependent masks were applied to the recorded images (*SI Appendix*, Fig. S5 *A* and *B*). The off-switching kinetics at 0.05 kW/cm^2^ and 1 kW/cm^2^ were subsequently plotted against each other for a comparative analysis (*SI Appendix*, Fig. S5*C*).

The measurements in mammalian cells confirmed the kinetic measurements of PENELOPE in *E. coli* colonies. After about 4 ms in the dark, the switched-off proteins thermally relaxed back to 50% of their original brightness (*SI Appendix*, Fig. S6*A*). Using moderate illumination intensities of about 1 kW/cm^2^, a switching halftime of 70.9 µs and a residual fluorescence in the off state of 4.8% was reached (*SI Appendix*, Fig. S6 *B* and *C*). Higher light intensities did not increase the switching speed further. These parameters led to a three-step RESOLFT imaging protocol: On each pixel, i) few milliseconds without illumination for relaxation of PENELOPE to the on-state are followed by ii) an off-switching step with a donut shaped 660 nm light pattern of low intensity for a few hundred microseconds and iii) a fluorescence read-out step using a Gaussian shaped 660 nm light pattern with high intensity for a few microseconds.

Next, we tested whether chemical fixation alters the switching kinetics of PENELOPE. Mammalian cells stably expressing cytokeratin-18-PENELOPE were treated with 4% formaldehyde for 5 min, and the switching kinetics of the chemically fixed proteins were compared to the kinetics in living cells using a confocal microscope (*SI Appendix*, Fig. S6*D*). The switching halftime and residual fluorescence of PENELOPE were only slightly affected by fixation. This suggests that PENELOPE is the first genetically encoded RSFP shown to be compatible for RESOLFT microscopy of live as well as fixed mammalian cells.

### NIR RESOLFT Superresolution Microscopy with a Single Wavelength in Mammalian Cells.

To explore single-wavelength NIR RESOLFT imaging of chemically fixed cells, the cell line stably expressing cytokeratin-18-PENELOPE was used. The RESOLFT image was recorded with the following optimized sequence at every pixel: First, thermal relaxation without illumination for 5 ms, followed by 400 µs off-switching with a doughnut-shaped 660 nm light distribution (1.1 kW/cm^2^), and finally fluorescence readout for 10 µs with a regularly focused 660 nm laser beam (103 kW/cm^2^).

**Fig. 4.**
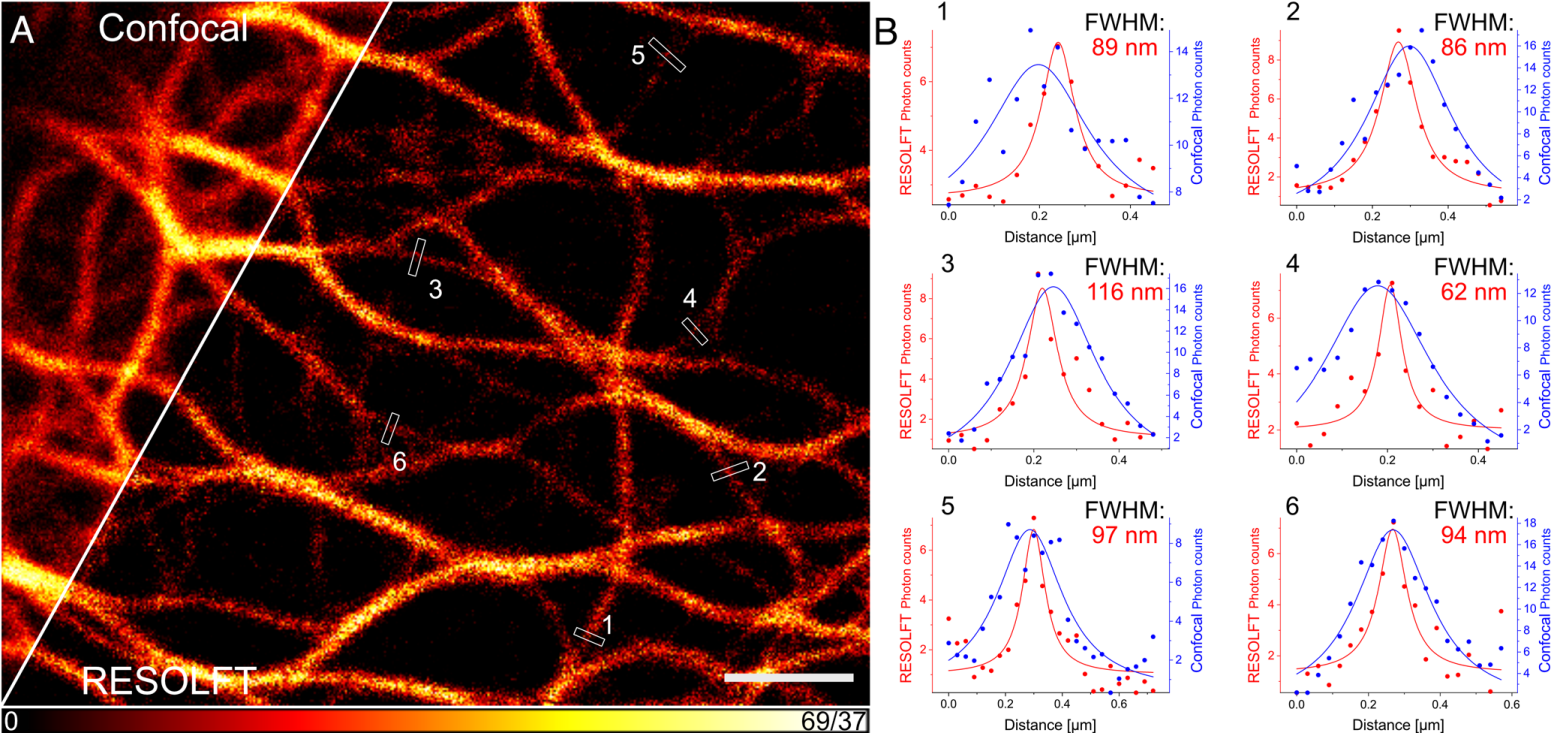
RESOLFT imaging of a formaldehyde-fixed HeLa cell stably expressing cytokeratin-18-PENELOPE. (*A*) Formaldehyde-fixed HeLa cells were imaged in a pixel step mode with 30 nm pixels. The confocal image and the RESOLFT image were recorded simultaneously. The total pixel dwell-time was 10.42 ms consisting of 5 ms thermal relaxation, a 10 μs step of 660 nm illumination with 103 kW/cm^2^ to record the confocal image, followed by 5 ms thermal relaxation, illumination with a 1.1 kW/cm^2^ doughnut-shaped 660 nm light distribution for off-switching for 400 μs and readout with a 103 kW/cm^2^ Gaussian-shaped 660 nm light distribution for 10 μs to record the RESOLFT image. 25 μM BV was added 2 h before imaging. (*B*) The presented line profiles are an average of three adjacent pixels. The data points were fitted with a Lorentzian function. (Scale bar: 2 μm.)

**Fig. 5.**
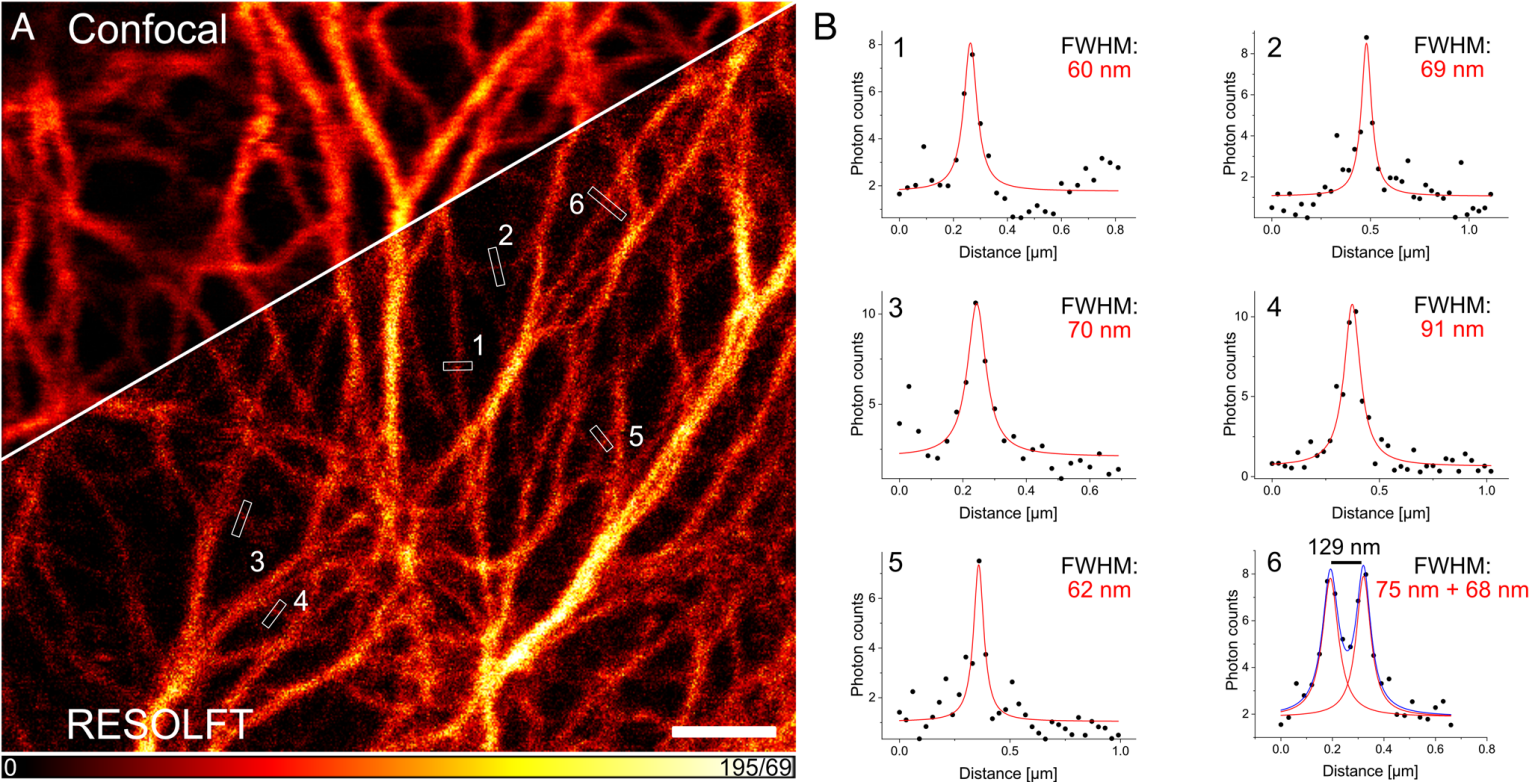
RESOLFT imaging of cytokeratin-18-PENELOPE fusion constructs in transfected live HeLa cells. (*A*) Live HeLa cells were imaged in a pixel step mode with 30 nm pixels. The confocal image was recorded simultaneously with the RESOLFT image. The total pixel dwell time consisted of 5 ms thermal relaxation (to facilitate the transfer into the on-state) and a 15 μs 660 nm illumination with 82 kW/cm^2^ to record the confocal image, followed by 5 ms thermal relaxation, illumination with a 1.1 kW/cm^2^ doughnut-shaped 660 nm off-switching light distribution for 1.5 ms and readout with an 82 kW/cm^2^ Gaussian-shaped 660 nm light distribution for 15 μs to record the RESOLFT image. 25 μM BV was added 2 h before imaging. (*B*) The presented line profiles were measured manually at selected positions and averaged across three adjacent pixels. The data points were fitted with a Lorentzian function. (Scale bar: 2 μm.)

A comparison between the RESOLFT image and the corresponding diffraction-limited confocal image reveals the attained higher resolution in the RESOLFT recording (Fig. 4). In order to estimate the improvement in resolution, we relied on averaged line profiles across individual cytokeratin filaments. Although the analysis of averaged line profiles has inherent limitations, as it depends on multiple factors including the structure and the local signal-to-noise ratio, it provides Full Width at Half Maximum (FWHM) numbers reflecting the achieved resolution in the raw images. In case of the RESOLFT images, averaged line profiles across individual cytokeratin filaments exhibited a typical FWHM of ~60 to 110 nm (Fig. 4), suggesting that RESOLFT imaging improved the resolution of the cytokeratin filaments by a factor of up to four compared to the corresponding confocal image. Next, we aimed at performing NIR RESOLFT microscopy using PENELOPE in living cells (Fig. 5). The RESOLFT image was recorded using the following recording scheme at each pixel: starting with thermal relaxation in the absence of illumination (5 ms), then off-switching using a 660 nm doughnut-shaped light distribution at an intensity of 1.1 kW/cm^2^ for 1.5 ms, and lastly, performing a 15 µs readout using a conventionally focused 660 nm light beam at an intensity of 82 kW/cm^2^. Fluorescence was collected at 690 to 750 nm. Using this recording scheme, we performed RESOLFT imaging of living cells expressing cytokeratin-18-PENELOPE with only one laser of 660 nm for switching and excitation. We consistently achieved FWHMs of about 60 nm measured at individual keratin filaments. Thus, single-wavelength NIR RESOLFT superresolution microscopy in living cells was established and allowed resolution of adjacent filaments as close as 129 nm apart (Fig. 5), impossible using diffraction-limited microscopy.

## Discussion

The spectral limitations of conventional GFP-like RSFPs restricted RESOLFT superresolution microscopy to the visible spectrum, but PENELOPE opens the door to NIR RESOLFT microscopy. Despite their different evolutionary origins, PENELOPE shares features with known RSFPs from the GFP family that are necessary for the application in RESOLFT superresolution microscopy: a high resistance against switching fatigue, low residual fluorescence in the nonfluorescent off-state, and fast off-switching kinetics (41). In contrast to RSFPs from the GFP family, PENELOPE does not require potentially harmful UV-light for switching (17) and withstands chemical fixation while maintaining light-dependent off-switching and light-independent thermal relaxation to the fluorescent on-state.

In the established NIR RESOLFT imaging scheme, PENELOPE’s thermal relaxation, which induced a millisecond-scale recovery from a metastable nonfluorescent off-state into a fluorescent on-state, was harnessed instead of light-driven on-switching, which is typically used in RESOLFT imaging with other RSFPs. The timescales of fluorescence recovery, by thermal relaxation, for other RSFPs are in the range of minutes to hours (10, 42, 43), which rendered this mode of imaging previously impossible.

The single wavelength imaging regime reduces the applied light intensity to just 1.275 kJ/cm^2^ for imaging living Hela cells. However, as implemented in this study, it requires comparatively long pixel dwell times and therefore about ten times longer acquisition times compared to the widely used rsEGFP2 (9). This limitation might be addressed by screening for FPs exhibiting a further shortened thermal relaxation time or an enhanced sensitivity to bathochromic on-switching wavelengths, in order to use fast light-driven on-switching. Ultimately, a parallelized RESOLFT imaging scheme (12, 44, 45) is likely to be the most efficient approach for utilizing probes such as PENELOPE for low-light RESOLFT imaging in the near infrared spectrum.

## Methods

### *E. coli* Expression Systems.

Three different *E. coli* strains were used during this study. The *E. coli* strain DH5α (MAX Efficiency DH5αF’IQ Competent Cells; Life Technologies, CA) was used for molecular cloning. The *E. coli* strain TOP10 (TOP10 Electrocomp Kits; Invitrogen, Waltham, MA) was used for plasmid amplification, and the *E. coli* strain BL21AI was used for fluorescent protein expression (BL21-AITM One ShotTM Chemically Competent *E. coli*, Thermo Fisher Scientific, Waltham, MA).

In preparation for fluorescent protein expression, the *E. coli* BL21AI cells were first transformed with a heme-oxygenase expressing pWA23h-plasmid (25) (kindly provided by Vladislav V. Verkhusha, Albert Einstein College of Medicine, New York, United States). This allowed Rhamnose-induced heme oxygenase expression for the increased degradation of heme to BV, Fe^3+^, and CO in addition to natural *E. coli* heme degradation. The hmuO gene codes for the heme-oxygenase of Bradyrhizobium ORS278. These pWA23h-plasmid expressing BL21AI-cells were then used for fluorescent protein expression required for protein purification, screening, and analysis of switching characteristics.

The underlying expression system for all *E. coli*–related experiments was the pBAD/His-B backbone containing pBAD-mKalama1 expression vector, which was kindly provided by Robert Campbell (Addgene Plasmid # 14892). Fluorescent protein expression was controlled by an arabinose-inducible araBAD promoter. An N-terminally added and TEV-protease cleavable 6× His-tag allowed protein purification. Restriction enzyme cloning into this vector required insert amplification and subsequent digestion with restriction enzymes EcoRI/SalI. The digested insert was ultimately ligated into the EcoRI/XhoI digested expression vector.

### Protein Expression and Purification.

For protein expression, proteins of interest were cloned in a pBad expression plasmid and transformed into BL21-AI *E. coli* (Invitrogen, Carlsbad, USA), which were already transformed with the heme oxygenase expressing pWA23h plasmid (as described in the previous section). Bacterial colonies were grown on rectangular RFA agar plates containing 0.02% arabinose and 0.02% rhamnose along with 50 µM FeCl_3_ and 100 µM δ-aminolevulinic acid (ALA) (Sigma-Aldrich, St. Louis, MO). Protein expression was performed at 37 °C for 18 to 24 h.

Following the expression, cells were collected via cell scraping in 2 mL binding buffer (20 mM phosphate, 500 mM NaCl, and 20 mM imidazole, pH 7.4). Next, the cell suspension was incubated with 10 µg/mL lysozyme (Serva electrophoresis, Heidelberg, Germany) on ice for 5 h. Following lysozyme treatment, a complete protease inhibitor (Roche, Basel, Switzerland) was added, incubated for 10 min on ice and subsequently frozen and thawed five consecutive times with liquid nitrogen and lukewarm water. Finally, cell lysate was supplemented with 0.5 µl benzonase (Thermo Fisher Scientific, MA), and protein solution was separated from debris by centrifugation 4 °C and 21,000 rcf for 4 h. Isolation of proteins of interest from supernatant was performed by Ni-NTA affinity chromatography (His SpinTrap Kit, GE Healthcare, Little Chalfont, GB) according to the manufacturer’s protocol. After purification, the protein concentration was determined using the Bio-Rad Bradford assay (Hercules, CA). Purified protein was taken up in the protein buffer consisting of 100 mM Tris-HCl, 150 mM NaCl, pH 7.5.

### Constructs for Expression in Mammalian Cells.

To evaluate the suitability of PENELOPE as a protein fusion tag for confocal and superresolution microscopy, PENELOPE was linked to variety of proteins of interest, namely: cytokeratin-18, vimentin, microtubules (α-tubulin), microtubule ends (EB3), zyxin, F-actin (Lifeact), OMP25, Cox8a, nuclear pore complex protein 50 (Nup50), Histones (H2B), Lysosomes (LAMP1), and Peroxisomes.

For the generation of a human cytokeratin-18 fusion construct, the PENELOPE coding sequence was amplified with the primers 5’ACGGTACCGCGGGCCCGGGATCCACCGGTCGCCACCATGTCCCGTGACCCTCTCCCCT3’ and 5’AGCTGTGCGGCCGCTCAACTCTGGCGAAAGGCGGCAAC3’. The PCR product was exchanged with the TagRFP coding sequence in pTagRFP-Keratin18 (Evrogen, Moscow, Russia) using KpnI and NotI, resulting in pKrt18-PENELOPE.

To target vimentin, the coding sequence of PENELOPE was amplified using the primers 5’TCCCCCGGGCGCCACCATGTCCCGTGACCCTCTCCCCT3’ and 5’TCAGCGGCCGCTCAACTCTGGCGAAAGGCGGCAA3’. The PCR product was exchanged with the mKate2 coding sequence in pmKate2-Vimentin (Evrogen, Moscow, Russia) using AgeI/XmaI and NotI, resulting in pVIM-PENELOPE.

To target tubulin, the coding sequence of PENELOPE was amplified using the primers 5’ TCCGCTAGCGCTACCGGTCGCCACCATGTCCCGTGACCCTCT3’ and 5’ CACTCGAGATCTGAGTCCGGAACTCTGGCGAAAGGCGG3’. The PCR product was exchanged with the mEGFP coding sequence in pEGFP-αTubulin (Clontech) using NheI and BglII, resulting in pPENELOPE-αTubulin.

To label microtubule filament ends, the coding sequence for human microtubule-associated factor EB3 had previously been PCR amplified from ptagRFP657-EB3 and inserted into TagRFP-N (Evrogen, Moscow, Russia) with restriction enzymes SalI and BamHI. The coding sequence of PENELOPE was amplified using the primers 5’TCCCCCGGGCGCCACCATGTCCCGTGACCCTCTCCCCT3’ and 5’CACTCGAGATCTGAGTCCGGAACTCTGGCGAAAGGCGG3’. The PCR product was exchanged with the TagRFP coding sequence using AgeI/XmaI and NotI, resulting in pEB3-PENELOPE.

To target zyxin, the coding sequence of PENELOPE was amplified using the primers 5’TCCCCCGGGCGCCACCATGTCCCGTGACCCTCTCCCCT3’ and 5’TTCTGCGGCCGCACTCTGGCGAAAGGCG3’. The PCR product was exchanged with the mMaple3 coding sequence in pZyxin-mMaple3 (this plasmid was a gift from Xiaowei Zhuang, Addgene plasmid # 101151) using AgeI/XmaI and NotI, resulting in pZyxin-PENELOPE.

F-actin filaments were labeled indirectly with PENELOPE fused to LifeAct (ibidi GmbH, Gräfelfing, Germany). For the generation of a PENELOPE-LifeAct fusion construct, the PENELOPE coding sequence was amplified with the primers 5’ACGGTACCGCGGGCCCGGGATCCACCGGTCGCCACCATGTCCCGTGACCCTCTCCCCT3’ and 5’AGCTGTGCGGCCGCTCAACTCTGGCGAAAGGCGGCAAC3’. The PCR product was exchanged with the EGFP coding sequence in Lifeact-EGFP pcDNA3.1(+) (Evrogen, Moscow, Russia) using KpnI and NotI, resulting in pLifeAct-PENELOPE.

To generate PENELOPE-OMP25 constructs targeting the mitochondrial outer membrane, the coding sequence of PENELOPE was amplified using the primers 5’TCCGCTAGCGCTACCGGTCGCCACCATGTCCCGTGACCCTCT3’ and 5’CACTCGAGATCTGAGTCCGGAACTCTGGCGAAAGGCGG3’. The PCR product was exchanged with the EGFP coding sequence in pEGFP-OMP25 (a kind gift from Joerg Bewersdorf, Yale School of Medicine, Connecticut, United States) using NheI and BglII, resulting in pPENELOPE-OMP25.

To generate Cox8a-PENELOPE constructs targeting the mitochondrial inner membrane, the coding sequence of PENELOPE was amplified using the primers 5’ TACGGATCCGATGTCCCGTGACCCTCTCCCCTT’ and 5’TCAGCGGCCGCTCAACTCTGGCGAAAGGCGGCAA3’. The PCR product was exchanged with the DsRed1 coding sequence in pDsRed1-Mito (a kind gift from Peter Lipp, Saarland University, Homburg, Germany) using BamHI and NotI, resulting in pCox8a-PENELOPE.

To target the nuclear pore complex via Nup50, the coding sequence of PENELOPE was amplified via PCR using the primers 5’TCCGCTAGCGCTACCGGTCGCCACCATGTCCCGTGACCCTCT3’ and 5’CACTCGAGATCTGAGTCCGGAACTCTGGCGAAAGGCGG3’. The Nup50 fusion construct was cloned by exchanging mEmerald with the PCR product via NheI and BglII in pmEmerald –Nup50 construct (this plasmid was a gift from Michael Davidson, Addgene plasmid # 54209).

For Histone 2B, EGFP was exchanged with PENELOPE via NheI and BglII restriction sites in the construct pEGFP-Hist1H2BN which was described in ref. 30.

To target lysosomes via the Lysosomal-associated membrane protein 1 (LAMP-1), the coding sequence of PENELOPE was amplified using the primers 5’TCCCCCGGGCGCCACCATGTCCCGTGACCCTCTCCCCT3’ and 5’TTCTGCGGCCGCACTCTGGCGAAAGGCG3’. The PCR product was exchanged with the rsCherryRev1.4 coding sequence in pLamp1-rsCherryRev1.4-N using AgeI/XmaI and NotI, resulting in pLamp1-PENELOPE.

To target peroxisomes, a plasmid expressing PENELOPE with the peroxisomal targeting sequence (PTS) at its C-terminus was generated as follows: The PTS was fused to the coding sequence of mEGFP by PCR using the primers 5′CGACGCTAGCATGGTGAGCAAGGGCG3′ and 5′AACAGGATCCCTACAGCTTGGACACTCGAGATCTGAGTCCGGACTTGTACAGCTCGTCCATGCC3′. Subsequently, this PCR product was exchanged with the coding sequence of pEGFP-Tub in pEGFP-Tub (BD Biosciences Clontech) using NheI and BamHI, resulting in pEGFP-PTS (as described in ref. 30). In the last step, the coding sequence of PENELOPE (amplified with primers 5’TCCGCTAGCGCTACCGGTCGCCACCATGTCCCGTGACCCTCT3’ and 5′AACAGGATCCCTACAGCTTGGACACTCGAGATCTGAGTCCGGACTTGTACAGCTCGTCCATGCC3′) was exchanged with the EGFP coding sequence using BglII and NheI, resulting in pPENELOPE-PTS.

### Establishment of Cells Stably Expressing PENELOPE-Keratin.

To establish a stable cell line expressing a PENELOPE fusion protein, a workflow established by Duportet et al. (described in ref. 46) was followed. This approach, which allows for incorporation of a single plasmid into the genome, lowered the expression heterogeneity often observed in transiently transfected cells. The establishment was performed in a HeLa cell line modified to have a CAG promoter upstream of a Bxb1 attP site. An integration plasmid containing a multiple cloning site and a fluorescent internal expression reference (mEGFP) was used to create the final integration-ready Keratin-PENELOPE plasmid. Stable integration was achieved by cotransfecting 1 μg of the integration-ready plasmid together with 1 μg of the plasmid pCAG-NLS-HA-Bxb1 (47). The plasmid pCAG-NLS-HA-Bxb1 was a gift from Pawel Pelczar (Addgene #51271) and encoded for the transient expression of the Bxb1 recombinase with a nuclear localization signal.

Transfected cells were kept in culture for 7 d. Following FACS analysis, fluorescent cells were single-cell sorted into 96-well plates and kept in culture for 21 d and then analyzed with a Cytation 3 Cell Imaging Multi-Mode Reader (BioTek, Winooski, VT) for correct localization under 640 nm light illumination.

### Mammalian Cell Culture.

HeLa (ATCC CCl-2) cells were cultivated in Dulbecco’s Modified Eagle’s Medium (DMEM-Medium: 4.5 g/L glucose, GlutaMAX, phenol red, 10% vol/vol FCS, 1 mM sodium pyruvate, 100 µg/mL streptomycin, 100 µg/mL penicillin) in T25 flasks for adherent cells as well as in 6-well plates for imaging and FACS analysis at 37 °C, 90% humidity, and 5% CO_2_. Transient transfection was conducted using the JetPrime transfection kit (Polyplus transfection, Illkirch, France) together with the respective expression plasmid. Approximately 2 h before FACS analysis or imaging, 25 µM BV per well was added to the medium.

### Spectral Characteristics.

All spectral measurements were performed with purified protein in 100 mM Tris-HCl, 150 mM NaCl, pH 7.5 or in vitro in living *E. coli* (PBS suspension). Absorption and emission spectra of purified protein were measured on a Varian Cary 4000 UV/vis spectrometer and a Varian Cary Eclipse fluorescence spectrometer (both Varian, Palo Alto, CA) in an ultra–micro fluorescence cell cuvette with a 1.5 mm light path (Hellma, Müllheim, Germany). Alternatively, the fluorescence of PENELOPE in *E. coli* suspension was measured with a HORIBA Fluorolog-3 in a 4 mm Thorlab absorption quartz cuvette and in front-face mode. Thermal recovery was investigated by absorption with a Cary 3500 Compact UV-Vis spectrometer with air-cooled Peltier temperature control. The EC of PENELOPE at 689 nm was calculated relative to free Biliverdin in solution (39,900 M^−1^ cm^−1^). The estimation of holoprotein:apoprotein was made by comparing the ratio of AbsMax(689)/A280 (total protein) to the ratio of εMax(689)/ε280 (r1 and r2 in *SI Appendix*, Table S1). All spectra were baseline corrected according to the Trp, Tyr, Phe content and normalized to the 280 nm absorption peak. Switching to the on- or off-state was performed in an ultra–micro fluorescence cell cuvette with a mercury-vapor lamp with a Brightline Fluorescence 661/20 filter (Semrock, NY) for off-switching (9.9 mW/cm^2^ measured behind the cuvette filled with Tris protein buffer) and an M780LP1 LED (Thorlabs, NJ, USA) for on-switching (18.7 mW/cm^2^) measured behind the cuvette filled with Tris protein buffer). Power was measured with a PM200 power meter equipped with an S170C sensor (Thorlabs, Newton, NJ).

### Fluorescence Quantum Yield.

Fluorescence quantum yield was determined either using a Quantaurus-QY system (Hamamatsu, Hamamatsu City, Japan) or by Alexey Chizhik using a nanocavity-based method (39). Both procedures used purified protein solution in standard protein buffer at pH 7.5.

### Switching Quantum Yield.

Pfr formation can be formulated as a reaction rate equation (Eq. **SI1**) for a certain irradiation wavelength, where $\Phi_{Pr-Pfr}^{irr}$ and $\Phi_{Pfr-Pr}^{irr}$ are the off- and on-switching quantum yields, respectively. $k_{Pfr-Pr}$ is the thermal recovery rate constant from the Pfr to the Pr form. $I_{Pr}^{abs}$ and $I_{Pfr}^{abs}$ are the absorbed light for the different protein forms at irradiation wavelength with $l_{irr}$ the optical path length and A the absorbance [$\frac{\left(1-10^{-A_{irr}}\right)}{A_{irr}}$ is also known as the photokinetics factor] considering the respective molar absorption coefficients $\varepsilon_{Pr}^{irr}$ and $\varepsilon_{Pfr}^{irr}$ and the photon flux density $I_{0}^{irr}$ (Eq. **SI2** and Eq. **SI3**) at the irradiation wavelength $\lambda_{irr}$.

[SI1] $$-\frac{d\left[\mathrm{Pr}\right]}{\mathrm{dt}}=\frac{d[\mathrm{Pr}]}{\mathrm{dt}}=\Phi_{\mathrm{Pr}-\mathrm{Pfr}}^{\mathrm{irr}}I_{\mathrm{Pr}}^{\mathrm{abs}}-\Phi_{\mathrm{Pfr}-\mathrm{Pr}}^{\mathrm{irr}}I_{\mathrm{Pfr}}^{\mathrm{abs}}-k_{\mathrm{Pfr}-\mathrm{Pr}}[\mathrm{Pfr}],$$

[SI2] $$\begin{array}{c} I_{\mathrm{Pr}}^{\mathrm{abs}}=I_{0}^{\mathrm{irr}}\frac{l_{\mathrm{irr}}\varepsilon_{\mathrm{Pr}}^{\mathrm{irr}}\left[\mathrm{Pr}\right]}{A_{\mathrm{irr}}}\left(1-10^{-A_{\mathrm{irr}}}\right)\\ \;=I_{0}^{\mathrm{irr}}\frac{\varepsilon_{\mathrm{Pr}}^{\mathrm{irr}}[\mathrm{Pr}]}{\varepsilon_{\mathrm{Pr}}^{\mathrm{irr}}[\mathrm{Pr}]+\varepsilon_{\mathrm{Pfr}}^{\mathrm{irr}}[\mathrm{Pfr}]}\left(1-10^{-l_{\text{irr}}\left(\varepsilon_{\text{Pr}}^{\text{irr}}[\text{Pr}]+\varepsilon_{\text{Pfr}}^{\text{irr}}[\text{Pfr}]\right)}\right), \end{array}$$

[SI3] $$\begin{array}{c} I_{\mathrm{Pfr}}^{\mathrm{abs}}=I_{0}^{\mathrm{irr}}\frac{l_{\mathrm{irr}}\varepsilon_{\mathrm{PFr}}^{\mathrm{irr}}\left[\mathrm{Pr}\right]}{A_{\mathrm{irr}}}\left(1-10^{-A_{\mathrm{irr}}}\right)\\ \;=\frac{\varepsilon_{\mathrm{Pfr}}^{\mathrm{irr}}[\mathrm{Pfr}]}{\varepsilon_{\mathrm{Pr}}^{\mathrm{irr}}[\mathrm{Pr}]+\varepsilon_{\mathrm{Pfr}}^{\mathrm{irr}}[\mathrm{Pfr}]}\left(1-10^{-l_{\text{irr}}\left(\varepsilon_{\text{Pr}}^{\text{irr}}[\text{Pr}]+\varepsilon_{\text{Pfr}}^{\text{irr}}[\text{Pfr}]\right)}\right). \end{array}$$

The determination of off-switching quantum yield was done by measuring the time (t) evolution of absorption spectra of Pr under irradiation and by calculating the differential quantum yield $\Phi_{d}^{irr}$ (48), i.e., the initial evolution (t = 0) of Pr concentration that can be measured using the absorbance at a probe wavelength for Pr (here at 680 nm, Eq. **SI4**). Indeed since the concentration of Pfr is negligible at t = 0 (i.e., after an extensive 780 nm illumination), the initial rate of change in absorbance dA_probe_/dt is given by Eq. **SI4**, which gives access to the P_r_-to-P_fr_ switching quantum yield at irradiation wavelength knowing precisely the photon flux density.

[SI4] $$\begin{array}{c} \Phi_{d}^{\mathrm{irr}}=-{\left(\frac{d\left[\mathrm{Pr}\right]}{\mathrm{dt}}\right)}_{t=0}\frac{1}{I_{0}^{\mathrm{irr}}\left(1-10^{-l_{\mathrm{irr}}\varepsilon_{\mathrm{Pr}}^{\mathrm{irr}}[Pr{]}_{t=0}}\right)}\\ \;=-{\left(\frac{dA_{\mathrm{probe}}}{\mathrm{dt}}\right)}_{t=0}\frac{1}{\varepsilon_{\mathrm{Pr}}^{\mathrm{probe}}l_{\mathrm{probe}}I_{0}^{\mathrm{irr}}\left(1-10^{-l_{\mathrm{irr}}\varepsilon_{\mathrm{Pr}}^{\mathrm{irr}}[Pr{]}_{t=0}}\right)}. \end{array}$$

The off-switching kinetics were measured on a custom-built microspectrometer experimental setup (49) that allows to measure the time evolution of absorption spectra. A Cobolt 06-MLD 638 nm laser diode was used for Pr to Pfr switching experiments, perpendicular to the white light probe beam. The Pr protein solution (230 µL, ≈10^−5^ M) was irradiated (10 × 2 mm cuvettes, 2 mm for 638 nm irradiation light with absorbance ≈0.1, and 1 cm for probe light) with the continuous diode laser paired with an aspheric condenser to increase the size and overfill the cuvette (>1 cm diameter). Seven different experiments at different photon flux density (between 1 and 20 µmol m^−2^s^−1^) were performed, with determination of $I_{0}^{638}$ using a diarylethene as a chemical actinometer (0.015 switching quantum yield at 638 nm) (48).

For the calculation of on-switching at 638 nm ($\Phi_{Pfr-Pr}^{638}$), as thermal recovery ($k_{Pfr-Pr}$) for protein in solution (in vitro) was slow enough to be neglected, on-switching at 638 nm was obtained from switching contrast [Pr]_0_/[Pr]_∞_ (Eq. **SI5**). The later was determined by comparing the initial fluorescence intensity $I_{fluo}^{0}$ to the photostationary state emission $I_{fluo}^{\infty }$ (excitation at 660 nm, emission detected at 720 nm)

[SI5] $$\frac{[\mathrm{Pr}{]}_{0}}{[\mathrm{Pr}{]}_{\infty }}=\frac{I_{\mathrm{fluo}}^{0}}{I_{\mathrm{fluo}}^{\infty }}=1+\frac{\varepsilon_{\mathrm{Pr}}^{638}\Phi_{\mathrm{Pr}-\mathrm{Pfr}}^{638}}{\varepsilon_{\mathrm{Pfr}}^{638}\Phi_{\mathrm{Pfr}-\mathrm{Pr}}^{638}}.$$

### Fluorescence Lifetime.

Fluorescence lifetime was determined using a Quantaurus-Tau fluorescence lifetime spectrometer (Hamamatsu, Hamamatsu City, Japan). For this, purified protein solution in protein buffer (100 mM Tris-HCl, 150 mM NaCl, as described for protein purification) at pH 7.5 was used.

### Seminative Polyacrylamide Gel.

2 to 4 µg purified protein solution in 100 mM TrisHCl, 150 mM NaCl, and 10% sucrose, pH7.5, was loaded onto a 15% polyacrylamide gel containing 0.1% sodium dodecyl sulfate. The gel was run for 1 h at 20 mA. Subsequently, the NIR fluorescence was detected using a gel imager (GE Healthcare, Chicago, IL) equipped with a 630 nm excitation and Cy5 filter set.

### Size Exclusion Chromatography.

An Äkta pure chromatography system equipped with a Superdex 200 Increase 10/300 column (GE Healthcare) was used for size exclusion chromatography. The purified protein solution was filtered using 0.2 µm spin columns (Sartorius, Göttingen, Germany). 250 µL at a concentration of 10 µM was added to the column and eluted at a flow rate of 0.75 mL/min with 100 mM TrisHCl, and 150 mM NaCl, pH7.5. The UV monitor U9-L (GE Healthcare) at 280 nm was used for protein detection. As standards, dTomato and mEGFP were measured. All runs were performed at 6 °C.

### Measurement of Laser Power.

For lasers used in conjunction with a 9420 series pulse generator (Quantum composers, Inc. Mt, USA), power measurement was performed in front of the objective at the sample plane with a PM200 power meter with an S170C sensor (ThorLabs, Newton, NJ). Laser powers of the automated custom-built screening microscope were measured in front of the objective (N PLAN L 20×/0.40, Leica Microsystems, Wetzlar, Germany) with a LabMax-TO laser power meter equipped with an LM-2 VIS sensor (Coherent, Santa Clara, CA).

### Imaging.

Imaging of transfected cells mounted in HEPES-buffered DMEM (HDMEM) without phenol red was performed approximately 24 h after transfection. Constitutively expressing mammalian cells were imaged 24 h after seeding. Cells were seeded on 18 mm glass coverslips No. 1.5H (Paul Marienfeld, Lauda-Königshofen, Germany) in 6-well plates (Sarstedt, Nümbrecht, Germany). 25 μM BV was added to the medium 2 h before imaging.

Confocal and RESOLFT imaging was performed on a commercial QUAD scanning fluorescence microscope (Abberior Instruments GmbH, Göttingen, DE) built around an Olympus microscope body (IX83, Olympus, Tokyo, JPN). The microscope was equipped with a UPLSAPO 1.4 NA 100× oil immersion objective (Olympus, Shinjuku, Japan) as well as a 660 nm continuous-wave laser (Cobolt, Solna, Sweden). This laser was split via a polarizing beam splitter (PBS) in two beams. The 660 nm doughnut-shaped laser beam was realized with an easy 3D module (Abberior Instruments GmbH, Göttingen, Germany). The NIR fluorescence (680 to 750 nm) light was focused through a pinhole on a silicon avalanche photodiode (Photon Counting Module SPCM-AQRH-13-FC, Excelitas Technologies, Waltham) for confocal and RESOLFT imaging. Microscope control and image acquisition were controlled by the software Imspector (Abberior Instruments GmbH, Göttingen, Germany). In addition to the utilized 660 nm lasers, further laser lines including the wavelengths 405 nm, 488 nm, 561 nm, and 785 nm (Cobolt, Solna, Sweden) were built into the microscope. These laser lines allowed for multicolor confocal imaging.

Confocal and RESOLFT images were imported in the image-processing package FIJI and displayed with the “Red Hot” lookup table. Images were exported as .tiff files. Images and filament intensity line profiles were measured averaging three adjacent pixels and were analyzed with the Fiji distribution of ImageJ (v1.53 h). Line profiles were fitted with a Lorentzian function in Origin Pro 2020 (OriginLab, Northampton, MA). All images shown are raw data, except stated otherwise. Switching kinetics in mammalian cells were recorded using a 9420 series pulse generator (Quantum composers, USA). Pulse generator measurements allowed laser control and detection with sub-μs temporal resolution. (OriginLab, USA).

### Switching Parameter Characterization.

Switching properties of all RSFP candidates including PENELOPE were measured in bacterial colonies with an automated custom-built screening microscope.

For evaluation of PENELOPE in mammalian cells, a stepwise scanning approach was used (3 µm × 3 µm region of interest with a Gaussian 660 nm laser), which was controlled with a 9420 series pulse generator (Quantum composers, Inc. Mt, USA) connected to the commercial QUAD scanning fluorescence microscope (Abberior Instruments GmbH, Göttingen, DE).

For the quantification of the switching speed and residual fluorescence as a function of the laser power, the illumination protocol consisted of a 20 ms nonillumination step to allow for complete thermal relaxation, followed by a 3 ms off-switching step with the Gaussian 660 nm laser with variable laser power. Off-switching data points were fitted with a gamma distribution. Bleaching was further investigated as each image was recorded three consecutive times, and the initial photon count of the last frame was divided by the photon count of the first frame.

The protocol for quantifying fluorescence recovery as a function of allowed thermal relaxation time included two illumination steps with a Gaussian 660 nm laser, which were interrupted by a time-variable nonillumination step. The ratio between the first value of the final readout illumination step and last value of the initial off-switching step was plotted against the duration of this time-variable nonillumination step (as time allowed for thermal relaxation).

## Supplementary Material

Appendix 01 (PDF)

## Data Availability

The raw data that support the findings of this study are available at Zenodo (50) (DOI: 10.5281/zenodo.17396934). All other data are included in the manuscript and/or *SI Appendix*.
