# Supplementary material for: The near-infrared bacteriophytochrome-derived fluorescent protein PENELOPE enables RESOLFT superresolution microscopy
Source: Proc Natl Acad Sci U S A. 2025 Nov 24;122(48):e2504748122. doi: 10.1073/pnas.2504748122 (PMC12685048; doi:10.1073/pnas.2504748122)
Supplement: Supplementary file 1 — Appendix 01 (PDF) [file pnas.2504748122.sapp.pdf]

Supplementary information to:

The near-infrared bacteriophytochrome-derived fluorescent protein PENELOPE enables RESOLFT super-resolution microscopy

Daniel Stumpf[1], Nickels Jensen[1], Cédric Mittelheisser[2], Jan Keller-Findeisen[1,3], Alexey I. Chizhik[4], Maria Kamper[1], Timo Diekmann[1], Florian Habenstein[1], Isabelle Jansen[1], Jörg Enderlein[4,5], Michel Sliwa[2,6], Kaushik Inamdar[1,7], Stefan W. Hell[1,5], Stefan Jakobs[1,3,5,7]*

[1] Department of NanoBiophotonics, Max Planck Institute for Multidisciplinary Sciences, 37077 Göttingen, Germany
[2] Université de Lille, CNRS, UMR 8516, LASIR, Laboratoire de Spectrochimie Infrarouge et Raman, 59000 Lille, France
[3] Fraunhofer Institute for Translational Medicine and Pharmacology ITMP, Translational Neuroinflammation and Automated Microscopy TNM, 37075 Göttingen, Germany
[4] Institute of Physics, Georg August University, 37077 Göttingen, Germany
[5] Cluster of Excellence "Multiscale Bioimaging: from Molecular Machines to Networks of Excitable Cells", University of Göttingen, Göttingen 37099, Germany.
[6] LOB, CNRS, INSERM, École Polytechnique, Institut Polytechnique de Paris, 91120 Palaiseau, France
[7] Clinic of Neurology, University of Göttingen, 37075 Göttingen, Germany

*Corresponding author: sjakobs@gwdg.de

	Dr-CBD$_{mono}$ (W3)	PENELOPE
$\lambda^{max}_{Soret-band}$ [nm]	392	391
λ^{max}_{Q-band} [nm]	696	689
A_{280}	0.208	0.219
$A^{max}_{Soret-band}$	0.086	0.108
A^{max}_{Q-band}	0.150	0.234
$r_1 = A^{max}_{Q-band}/A_{280}$	0.72	1.07
ε_{280} (BioExpasy) [$M^{-1}.cm^{-1}$]	32,555	36,565
$\varepsilon^{max}_{Q-band}$ (free BV method) [$M^{-1}.cm^{-1}$]	67,500	86,100
$r_2 = \varepsilon^{max}_{Q-band} / \varepsilon_{280}$	2.1	2.4
holo:apo (r1/r2)	1:2.9	1:2.2

Table S1: P_r molar absorption coefficients and calculation of the holoprotein:apoprotein proportions.

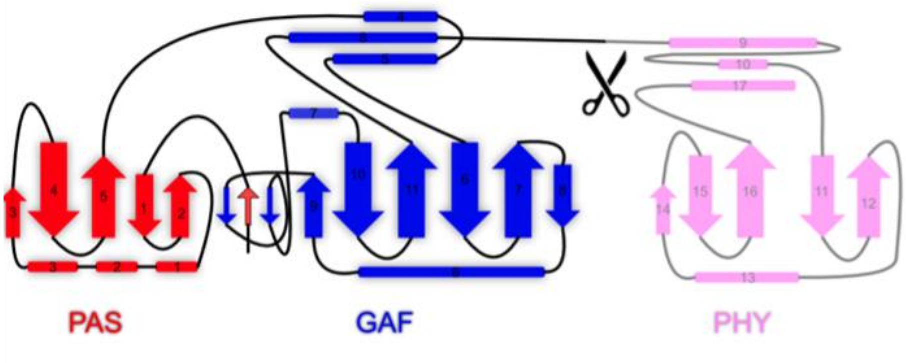

Figure S1: Schematic representation of the secondary structure of the bacteriophytochrome photosensory module (PSM) of *Deinococcus radiodurans*, with the PAS domain in red, GAF domain in blue and the PHY domain in magenta. For the development of PENELOPE, the PHY domain was truncated. The chromophore binding domain (CBD) consists of the PAS and GAF domains.

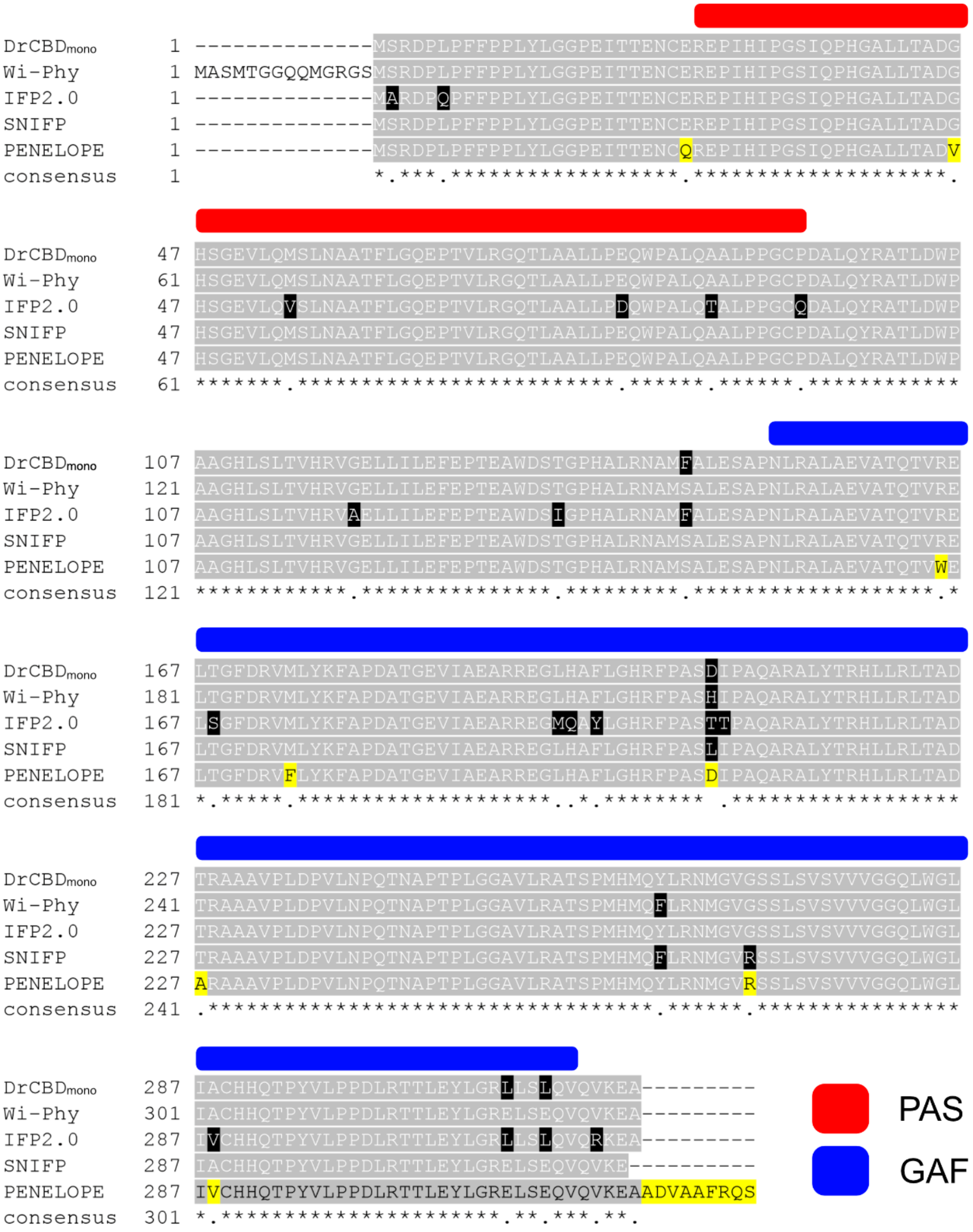

Figure S2: **Sequence alignment of *D. radiodurans*-derived constitutively fluorescent FPs with PENELOPE.** The amino acid sequence of PENELOPE aligned with the CBD domains of *D. radiodurans* (Dr-CBD), Wi-Phy, IFP2.0 and SNIFP. Amino acids of PENELOPE differing from the consensus sequence of its predecessors are highlighted in yellow. Amino acids of the other FPs differing from the overall consensus sequence are highlighted in black.

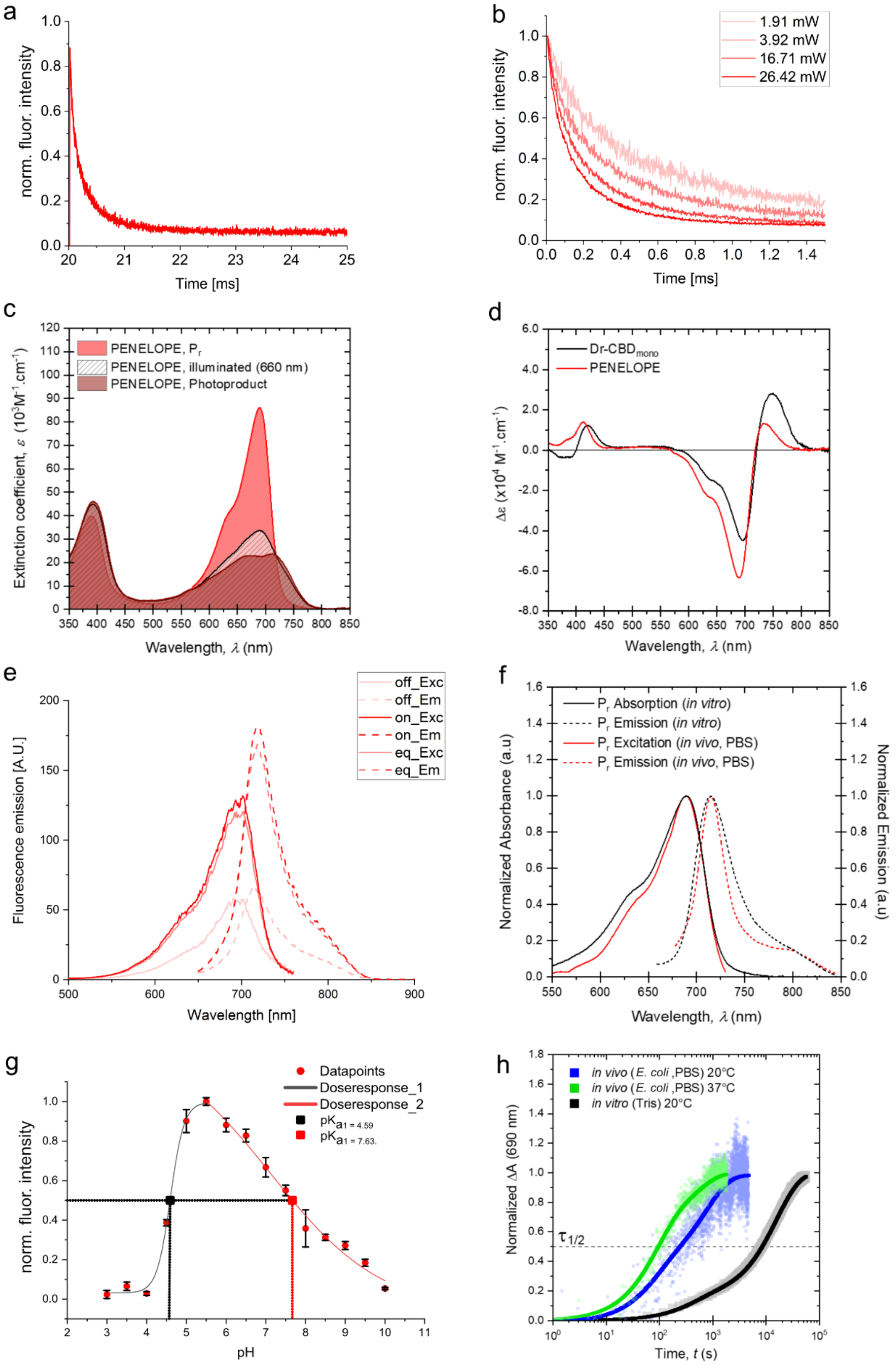
a
norm. fluor. intensity
Time [ms]
b
1.91 mW
3.92 mW
16.71 mW
26.42 mW
norm. fluor. intensity
Time [ms]
c
PENELOPE, Pr
PENELOPE, illuminated (660 nm)
PENELOPE, Photoproduct
Extinction coefficient, ε (10³M⁻¹.cm⁻¹)
Wavelength, λ (nm)
d
Dr-CBDmono
PENELOPE
Δε (x10⁴ M⁻¹.cm⁻¹)
Wavelength, λ (nm)
e
off_Exc
off_Em
on_Exc
on_Em
eq_Exc
eq_Em
Fluorescence emission [A.U.]
Wavelength [nm]
f
Pr Absorption (in vitro)
Pr Emission (in vitro)
Pr Excitation (in vivo, PBS)
Pr Emission (in vivo, PBS)
Normalized Absorbance (a.u)
Normalized Emission (a.u)
Wavelength, λ (nm)
g
Datapoints
Doseresponse_1
Doseresponse_2
pKa1 = 4.59
pKa1 = 7.63.
norm. fluor. intensity
pH
h
in vivo (E. coli ,PBS) 20°C
in vivo (E. coli ,PBS) 37°C
in vitro (Tris) 20°C
τ1/2
Normalized ΔA (690 nm)
Time, t (s)

Figure S3: Switching kinetics and spectral characterization of PENELOPE. (a) Single fluorescence traces of the first off-switching step of Figure 1f. (b) Zoom-in of single fluorescence traces of the off-switching step at different laser powers of Figure 1c. (c) Extinction coefficient spectra of PENELOPE in the ensemble pure on- and off-photostationary state. Pure off-state is calculated based on and off photostationary-state spectra and on-switching contrast (d) Absorption difference spectrum of Dr-CBD$_{mono}$ and PENELOPE. The difference spectra were generated by subtracting the extinction coefficient spectrum of the on-switched protein solution from the extinction coefficient spectrum of the pure off-switched protein solution. (e) Excitation and emission spectra of PENELOPE. Spectra were recorded at pH 7.5 with detection at 780 nm or excitation at 620 nm, respectively. Initial measurements were performed on the equilibrated sample. (f) Excitation and emission spectra of PENELOPE *in cellulo*. Spectra were recorded in living *E. coli* suspended in PBS with detection in front-face mode at 780 nm and excitation at 640 nm, respectively. (g) pH-dependent absorption and fluorescence emission spectra of PENELOPE. Fluorescence emission was measured with a 650/19 nm excitation and a 700/19 nm detection filter and results are depicted as a function of pH. (h) Absorption recovery (attributable to thermal relaxation) measured at 20°C (purified protein and *E. coli*) and 37 °C (*E. coli*).

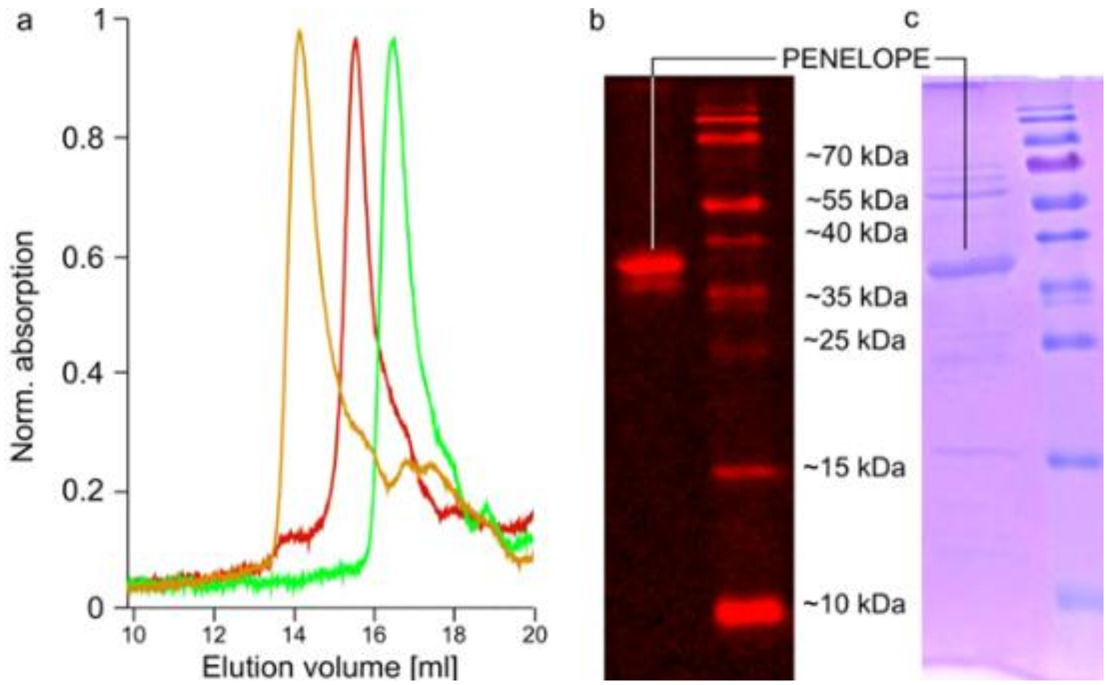

Figure S 4: Size exclusion chromatography and semi-native polyacrylamide gel electrophoresis (PAGE). (a) The absorption at 280 nm was detected after size exclusion chromatography (SEC) of protein samples with 10 µM concentration. SEC was performed at 6°C. dTomato (orange) and mEGFP (green) were used as dimeric and monomeric GFP markers, PENELOPE (red) elutes predominantly in the monomeric form. (b) Fluorescence image of PENELOPE in semi-native PAGE. (c) shows the same gel as presented in (b) with a Coomassie blue staining. PageRuler Prestained Protein Ladder was used as a molecular weight marker.

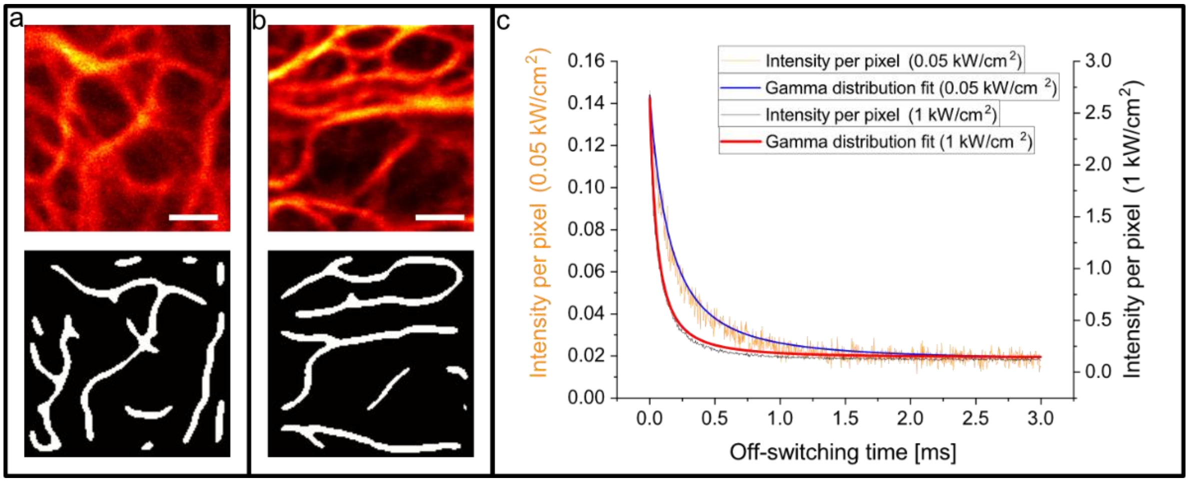

Figure S5: Intensity dependence off-switching curves recorded on live mammalian cells. (a, b) Confocal images of live mammalian cells expressing cytokeratin-18-PENELOPE and applied structure masks. a) was recorded with 0.05 kW/cm^2 and b) with 1 kW/cm^2. Shown are raw data. Scale bars: 1µm. (c) Comparative analysis of off-switching data at 0.05 kW/cm^2 (yellow), left Y-axis, and 1 kW/cm^2 (black), right Y-axis. A gamma fit was applied to the raw off-switching data for 0.05 kW/cm^2 (blue) and 1 kW/cm^2 (red).

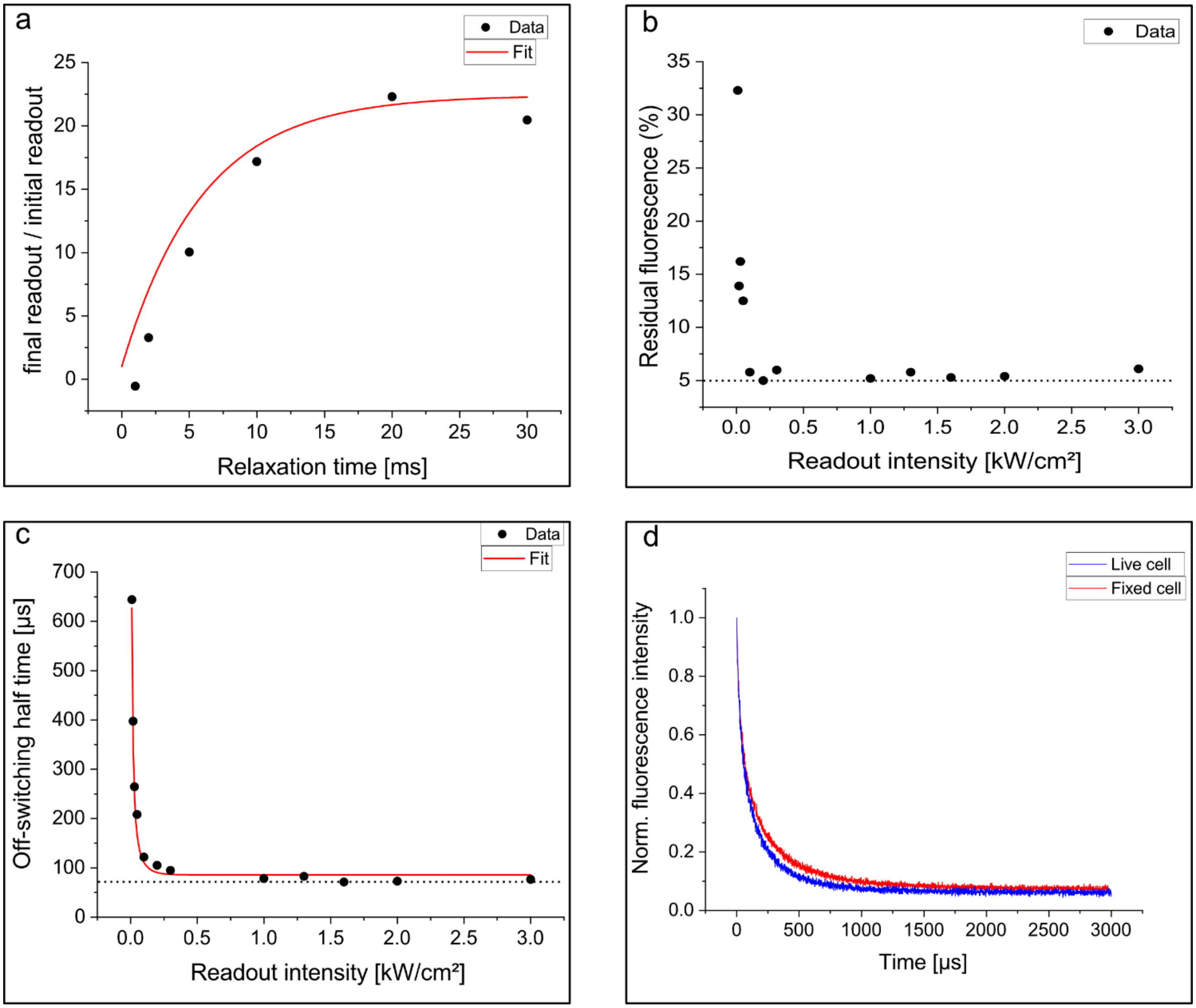

Figure S6: Kinetics analysis in mammalian cells using a confocal microscope. (a) Recovered fluorescence brightness as a function of relaxation time. The protocol consisted of repeated application of a 0.21 kW/cm^2 660 nm Gaussian-shaped laser for 3 ms. Between each repetition, a varying time (1-30 ms) for thermal relaxation was implemented, and final readout intensities at the end of the time intervals were recorded and plotted as a ratio with the initial intensities after off-switching. (b) Residual fluorescence normalized to the initial fluorescence (100%) as a function of readout laser intensities. The protocol consisted of the application of a 660 nm Gaussian-shaped laser with laser powers in the range of 0.01 kW/cm^2 to 3 kW/cm^2 for 3 ms for each readout. Minimum residual level is indicated by dashed line. (c) Off-switching half time as a function of laser intensities. The protocol consisted of the application of a 660 nm Gaussian-shaped laser with laser powers in the range of 0.01 kW/cm^2 to 3 kW/cm^2 for 3 ms for each readout. Minimum residual level is indicated by dashed line. Data shown for (a), (b) and (c) are based on experiments performed on a predefined area of 16 µm^2 in live cells. (d) Representative raw data off-switching curves of PENELOPE-cytokeratin18 recorded using 0.21 kW/cm^2 on live and fixed mammalian cells.
